# College News

**Published:** 1895-06

**Authors:** 


### COLLEGE NEWS.

Ohio Veterinary College Exercises.—The annual commencement exercises were held on March 18th at the college. Professor Thomas King, Dean of the College, addressed the graduating class on "Veterinary Medicine." Professor J. Shaller, M.D., also addressed the students. The valedictory address was delivered by Frederick Keebler, after which the diplomas were delivered by W. W. Symmes, with appropriate remarks.

The following are the list of successful graduates: J. S. Ellis, A. W. Weir, J. A. Wynn, Louis Cook, Horace Bradley, J. A. Meager, W. A. Aixby, E. O. Hess, of Cincinnati; and C. D. Werley, of Albany, Pennsylvania.

The United States College of Veterinary Surgeons is out with its announcement for the college year of 1895 and 1896, announcing the change to a two years' course. In addition it makes the following announcement relative to the degrees conferrred:

"Upon completion of the course and satisfactory passing of the examinations, as prescribed by the faculty, the student is allowed to present himself for the trustees' examination, and if found to be qualified the degree of Doctor of Veterinary Science (D.V.S.) will be granted, and also admitting the student to membership of the United States College of Veterinary Surgeons (M.U.S.C.V.S.)

"The degree of Fellowship of the United States College of Veterinary Surgeons (F.U.S.C.V.S.) will be granted to graduates of this college and to those of other colleges who have been in actual practice for five years, and who have been a credit to the profession and to their Alma Mater."

